# Cholesterol-Induced Hepatic Inflammation Does Not Underlie the Predisposition to Insulin Resistance in Dyslipidemic Female LDL Receptor Knockout Mice

**DOI:** 10.1155/2015/956854

**Published:** 2015-02-28

**Authors:** Nanda Gruben, Anouk Funke, Niels J. Kloosterhuis, Marijke Schreurs, Fareeba Sheedfar, Rick Havinga, Sander M. Houten, Ronit Shiri-Sverdlov, Bart van de Sluis, Jan Albert Kuivenhoven, Debby P. Y. Koonen, Marten H. Hofker

**Affiliations:** ^1^Molecular Genetics Section, Department of Pediatrics, University Medical Center Groningen, University of Groningen, Antonius Deusinglaan 1, 9713 AV Groningen, Netherlands; ^2^Center for Liver, Digestive and Metabolic Diseases, Department of Pediatrics, University Medical Center Groningen, University of Groningen, Hanzeplein 1, 9713 GZ Groningen, Netherlands; ^3^Academic Medical Center, Laboratory Genetic Metabolic Diseases, Meibergdreef 9, 1105 AZ Amsterdam, Netherlands; ^4^Department of Molecular Genetics, Maastricht University, P.O. Box 616, 6200 MD Maastricht, Netherlands

## Abstract

Chronic inflammation is considered a causal risk factor predisposing to insulin resistance. However, evidence is accumulating that inflammation confined to the liver may not be causal to metabolic dysfunction. To investigate this, we assessed if hepatic inflammation explains the predisposition towards insulin resistance in low-density lipoprotein receptor knock-out (*Ldlr* ^−/−^) mice. For this, wild type (WT) and *Ldlr* ^−/−^ mice were fed a chow diet, a high fat (HF) diet, or a high fat, high cholesterol (HFC) diet for 2 weeks. Plasma lipid levels were elevated in chow-fed *Ldlr* ^−/−^ mice compared to WT mice. Although short-term HF or HFC feeding did not result in body weight gain and adipose tissue inflammation, dyslipidemia was worsened in *Ldlr* ^−/−^ mice compared to WT mice. In addition, dyslipidemic HF-fed *Ldlr* ^−/−^ mice had a higher hepatic glucose production rate than HF-fed WT mice, while peripheral insulin resistance was unaffected. This suggests that HF-fed *Ldlr* ^−/−^ mice suffered from hepatic insulin resistance. While HFC-fed *Ldlr* ^−/−^ mice displayed the anticipated increased hepatic inflammation, this did neither exacerbate systemic nor hepatic insulin resistance. Therefore, our results show that hepatic insulin resistance is unrelated to cholesterol-induced hepatic inflammation in *Ldlr* ^−/−^ mice, indicating that hepatic inflammation may not contribute to metabolic dysfunction per se.

## 1. Introduction

Obesity is linked to many deleterious health consequences, including insulin resistance, type 2 diabetes (T2D) and the metabolic syndrome, a group of metabolic risk factors predisposing to T2D, and cardiovascular disease. Low-grade, chronic inflammation is considered as one of the most important mechanisms explaining the etiology of insulin resistance, T2D, and the metabolic syndrome [1]. However, evidence is accumulating that inflammation when confined to the liver may not be causal to metabolic dysfunction in obesity (for review see [2]). For instance, we recently demonstrated that hepatic inflammation does not contribute to insulin resistance in TNFR1-non-sheddable mice expressing a mutated TNFR1 ectodomain incapable of shedding and dampening the hepatic inflammatory response [3]. Furthermore, we showed that cholesterol-induced hepatic inflammation does not advance the development of systemic insulin resistance in male* Ldlr* ^−/−^ mice [4]. Being consistent with the outcome of these gain-of-function studies, others have shown that reduced hepatic inflammation not necessarily corresponds to enhanced insulin sensitivity in mice [5, 6], further indicating that factors other than hepatic inflammation may be causal in triggering insulin resistance.

Dyslipidemia, provoked by elevated plasma low-density lipoprotein (LDL) cholesterol and/or very low-density lipoprotein (VLDL) triglycerides levels and decreased high-density lipoprotein (HDL) cholesterol levels, may be such a causal factor in the development of insulin resistance [7]. Indeed, several studies have shown that dyslipidemia is an independent predictor of insulin resistance and T2D [8, 9]. Furthermore, lipid-lowering drugs have been shown to exhibit a positive effect on insulin sensitivity [10]. Nevertheless, dyslipidemia may also occur as a result of insulin resistance since hepatic lipogenesis, in contrast to gluconeogenesis, remains sensitive to insulin [11]. This leads to an increased production of plasma lipids due to overstimulation of insulin receptor pathways caused by hyperinsulinemia [11]. Hampered by the coexisting nature of dyslipidemia and obesity, its exact role in the etiology of insulin resistance therefore remains ill defined.

To further elaborate on these studies, we assessed the extent to which hepatic inflammation may explain the reported predisposition towards insulin resistance in dyslipidemic* Ldlr* ^−/−^ mice [12]. Furthermore, the rapid development of dyslipidemia [13, 14] and hepatic inflammation [14, 15] in these mice allows us to investigate their effect on insulin resistance before alterations in body weight occur. We opted to use female mice only as they confer a natural resistance against diet-induced obesity. This is of particular importance as adiposity drives the metabolic phenotype in most studies [2] and differences in insulin resistance have been shown to disappear after matching the mice for body weight [16]. Our data show that hepatic inflammation is not a causal factor in the development of hepatic insulin resistance in* Ldlr* ^−/−^ mice. Thus, in line with the studies mentioned above, but contrasting with the current dogma, our data do not support a role for hepatic inflammation in triggering insulin resistance.

## 2. Research Design and Methods

### 2.1. Animals and Diets

Age-matched (12–16 weeks) female* Ldlr* ^−/−^ mice on a C57BL/6J background [13] and wild type (WT) C57BL/6J mice were used for all experiments. Breeding pairs of* Ldlr* ^−/−^ mice were obtained from the Jackson Laboratory (Bar Harbor, ME, USA) and* Ldlr* ^−/−^ mice were bred in house. WT mice were purchased from Charles River (France). Mice were placed on a standard rodent chow diet, a high fat (HF) diet (containing 21% fat from milk butter and 0.02% cholesterol; Scientific Animal Food and Engineering, Villemoisson-sur-Orge, France), or a high fat, high cholesterol (HFC) diet (containing 21% fat from milk butter and 0.2% cholesterol; Scientific Animal Food and Engineering, Villemoisson-sur-Orge, France) for a period of 2 weeks with* ad libitum* access to food and water. Mice were housed individually and kept on a 12-hour light/12-hour dark cycle. Animals were anesthetized by isoflurane during all surgical operations and discomfort was minimized as much as possible. All animal experiments were approved by the ethics committee of the University of Groningen, which adheres to the principles and guidelines established by the European Convention for the Protection of Laboratory Animals.

### 2.2. Oral Glucose Tolerance Test and Intraperitoneal Insulin Tolerance Test

Mice were fasted for 6 hours before performing an oral glucose tolerance test (OGTT) or an insulin tolerance test (ITT). For the OGTT, a glucose bolus of 2 g/kg body weight of 20% glucose solution was given by gavage. For the ITT, an insulin dose of 0.3 U/kg body weight was injected intraperitoneally. Glucose levels were measured with a One Touch Ultra glucose meter before the test and at 15, 30, 60, 90, and 120 minutes after gavage or injection. In addition, fasted insulin levels were measured with an ultrasensitive insulin ELISA kit (Alpco Diagnostics, Salem, NH). The homeostasis model assessment of insulin resistance (HOMA-IR) was calculated from fasted insulin and glucose levels (fasted insulin (*μ*U/mL) × fasted glucose (mmol/liter)/22.5).

### 2.3. Hyperinsulinemic-Euglycemic Clamp

A hyperinsulinemic-euglycemic clamp (HIEC) was performed in conscious mice as described previously [17], with a modified protocol. In brief, mice were cannulated in the right* vena jugularis* to allow infusion of fluids for an HIEC. They were allowed to recover for 5–7 days before the HIEC was started. Before the HIEC, mice were fasted overnight for 9 hours and placed in experimental cages. Mice were infused at a rate of 0.10 mL/h for 4 hours with a solution containing 1% bovine serum albumin (Sigma Aldrich, Zwijndrecht, Netherlands), 30% glucose (3% [U-^13^C] glucose; 27% glucose), 110 mU/mL insulin (Actrapid, Novo Nordisk, Bagsvaerd, Denmark), and 40 *μ*g/mL somatostatin (Eumedica NV, Brussels, Belgium). To maintain euglycemia, a 30% glucose solution was infused (3% [U-^13^C] glucose; 27% glucose) via a second line and pump speeds were adjusted to the needs of the animal. Every 15 min, a blood sample was taken from the tail vein to determine plasma glucose levels, and every 30 minutes, a blood spot was collected on filter paper for gas chromatography-mass spectrometry (GC-MS) analysis.

### 2.4. Gas Chromatography-Mass Spectrometry Analysis and Calculations

Extraction of glucose from blood spots and GC-MS analysis of extracted glucose were performed according to van Dijk et al. [18]. Hepatic glucose production and metabolic clearance rate were calculated from GC-MS results using mass isotopomer distribution analysis as previously described [18].

### 2.5. Blood and Tissue Collection

The mice were fasted for 6 hours before being sacrificed. Tissues were rapidly removed, snap-frozen in liquid nitrogen, and stored at −80°C until further analysis. For histology, tissues were frozen or fixed in paraformaldehyde and embedded in paraffin. Blood was collected by a heart puncture and separated by centrifugation (3000 g, 10 min, 4°C). Plasma was decanted and frozen at −20°C.

### 2.6. Lipid Analysis

For hepatic triglyceride and cholesterol measurements, lipids were extracted from frozen livers according to the method of Bligh and Dyer [19]. Hepatic and plasma triglyceride and cholesterol levels were measured using commercially available kits from Roche (Mannheim, Germany). Hepatic free cholesterol levels were determined using a commercially available kit from DiaSys (Holzheim, Germany). For diacylglycerol (DAG) determination, lipids were extracted from frozen-crushed livers with MeOH : CHCl_3_ (1 : 2) and separated by thin-layer chromatography. Lipids were visualized with CuSO_4_ and quantified by comparing the density to a standard amount of DAG.

### 2.7. Immunoblot Analysis

Frozen tissues were homogenized for Western Blot analysis. Protein concentration was equalized and proteins were separated with SDS-PAGE and transferred to polyvinylidene difluoride membranes (GE Healthcare Life Sciences, Diegem, Belgium). Membranes were incubated overnight at 4°C with an antibody against pAKT (Ser473, Cell Signaling Technology, Leiden, Netherlands) or AKT (Cell Signaling Technology) in 5% bovine serum albumin. The following day, membranes were incubated with a secondary antibody containing horse-radish peroxidase (Goat-anti-rabbit: Bio-Rad, Veenendaal, Netherlands). To visualize the immune complex, membranes were treated with enhanced chemiluminescence reaction reagent and a picture was taken using Gel Doc XR+ Imaging system (Bio-Rad). Protein bands were analyzed using Image Lab 3.0.1 (Bio-Rad).

### 2.8. Gene Expression

To isolate RNA, liver biopsies were homogenized in Qiazol reagent and RNA was isolated according to the manufacturer's procedure (Qiagen, Venlo, Netherlands). Adipose tissue RNA was isolated using a commercially available kit (Qiagen). From liver and adipose tissue RNA, cDNA was synthesized for RT-PCR using a commercially available kit (Bio-Rad). RT-PCR was performed using Sybr Green Supermix (Bio-Rad). The following primer sequences were used for RT-PCR:* Tnfa*, forward CATCTTCTCAAAATTCGAGTGACAA, reverse TGGGAGTAGACAAGGTACAACCC;* Mcp1*, forward GCTGGAGAGCTACAAGAGGATCA, reverse ACAGACCTCTCTCTTGAGCTTGGT;* Cd68*, forward TGACCTGCTCTCTCTAAGGCTACA, reverse TCACGGTTGCAAGAGAAACATG;* Cd11b*, forward TCAGAGAATGTCCTCAGCAG, reverse TGAGACAAACTCCTTCATCTTC;* Ppia* forward TTCCTCCTTTCACAGAATTATTCCA, reverse CCGCCAGTGCCATTATGG.

### 2.9. Histological Analysis

Paraffin-embedded adipose tissue biopsies were sectioned at 4 *μ*m and stained with hematoxylin-eosin. Frozen liver sections of 5 *μ*m were used to stain for the macrophage marker CD68 (FA11, Abcam, Cambridge, UK).

### 2.10. Statistical Analysis

2-way ANOVA followed by Bonferroni posttests to correct for multiple testing was performed using Graph-Pad Prism 5.0 (San Diego, USA) to determine the differences between groups. 1-way ANOVA (Kruskal-Wallis) was used followed by Dunns multiple comparison test was used to determine the differences in phosphorylation of AKT between the groups. To ensure that the assumption of homogeneity of variances was met, this was tested before performing an ANOVA. *P* values < 0.05 were considered significant. Values are expressed as mean ± SEM and group sizes are indicated in the figure legends.

## 3. Results

### 3.1. Elevated Plasma Levels of Cholesterol and Triglycerides in* Ldlr* ^−/−^ Mice Fed an HF and HFC Diet

**Figure 1:**
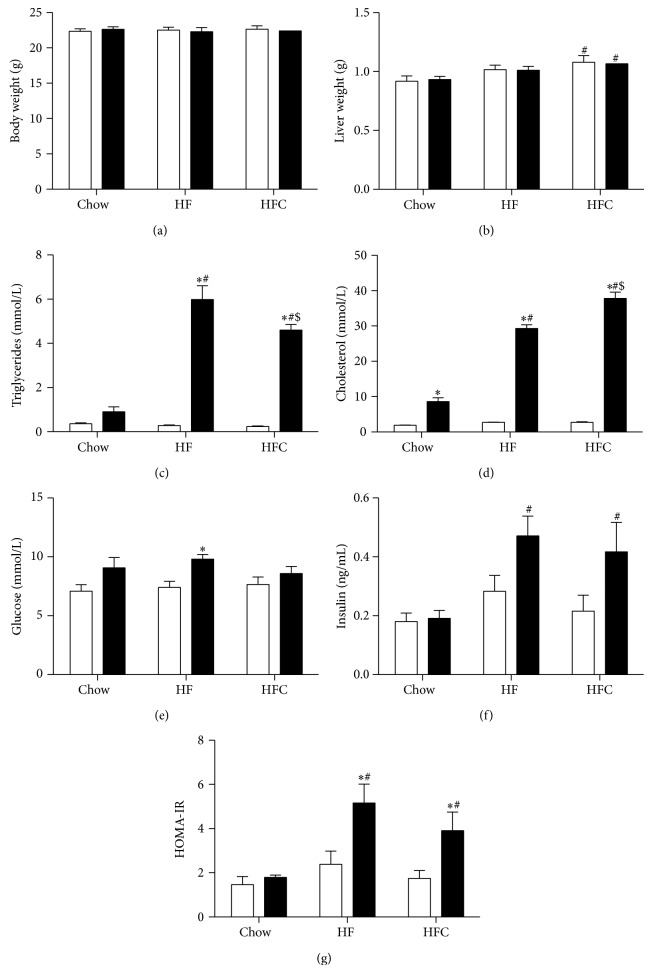
Circulating levels of lipids, glucose, and insulin in* Ldlr* ^−/−^ mice fed a chow, HF, or HFC diet for 2 weeks. Body weight (a) and liver weight (b) of WT and* Ldlr* ^−/−^ mice fed a chow, high-fat (HF), or high-fat cholesterol (HFC) diet were determined at time of sacrifice (*n* = 12). Plasma triglyceride (c), cholesterol (d), glucose (e), and insulin (f) levels were measured in blood obtained following a 6-hour fast (*n* = 5-6). (g) The homeostasis model assessment of insulin resistance (HOMA-IR) was calculated from fasted insulin and glucose levels (*n* = 6). Data are expressed as means ± SEM for WT mice (white bars) and* Ldlr* ^−/−^ mice (black bars). ^*^ *P* < 0.05 WT versus KO; ^#^ *P* < 0.05 HF, HFC versus chow; ^$^ *P* < 0.05 HF versus HFC.

Since dyslipidemia is an independent predictor of insulin resistance, we first assessed plasma lipid levels of WT and* Ldlr* ^−/−^ mice fed a chow, HF, or HFC diet for 2 weeks. Body weight (Figure 1(a)) did not differ between both genotypes; however, liver weight (Figure 1(b)) was slightly increased in both WT and* Ldlr* ^−/−^ mice fed an HFC-diet compared to chow-fed WT and* Ldlr* ^−/−^ mice (HFC versus chow, *P* < 0.05; HFC WT versus HFC KO, ns). Plasma triglyceride (Figure 1(c)) and cholesterol levels (Figure 1(d)) were significantly elevated in* Ldlr* ^−/−^ mice fed an HF- and HFC-diet compared to HF- and HFC-fed WT mice and* Ldlr* ^−/−^ mice fed a chow-diet (Figures 1(c) and 1(d)). Nevertheless, plasma triglyceride levels were significantly lower in HFC-fed* Ldlr* ^−/−^ mice compared to HF-fed* Ldlr* ^−/−^ mice (Figure 1(c)). In contrast, plasma cholesterol levels were significantly higher in HFC-fed* Ldlr* ^−/−^ mice compared to HF-fed* Ldlr* ^−/−^ mice (Figure 1(d)). No differences were observed in plasma FFA levels (data not shown). In addition, glucose levels were significantly elevated in* Ldlr* ^−/−^ mice fed an HF-diet compared to HF-fed WT mice (Figure 1(e)) and did not differ between chow- or HFC-fed* Ldlr* ^−/−^ mice (Figure 1(e)). However, insulin levels were significantly elevated in* Ldlr* ^−/−^ mice fed an HF and HFC diet compared to chow-fed* Ldlr* ^−/−^ mice (Figure 1(f)) but were not further increased by cholesterol addition to the HF diet (Figure 1(f)). In line with this, the HOMA-IR was significantly elevated in* Ldlr* ^−/−^ mice fed an HF and HFC diet compared to both HF- and HFC-fed WT mice and chow-fed* Ldlr* ^−/−^ mice (Figure 1(g)), confirming the reported predisposition towards insulin resistance in dyslipidemic* Ldlr* ^−/−^ mice [12]. HOMA-IR levels did not differ between HF- and HFC-fed* Ldlr* ^−/−^ mice (Figure 1(g)).

### 3.2. Increased Hepatic Inflammation in* Ldlr* ^−/−^ Mice Fed an HFC Diet

**Figure 2:**
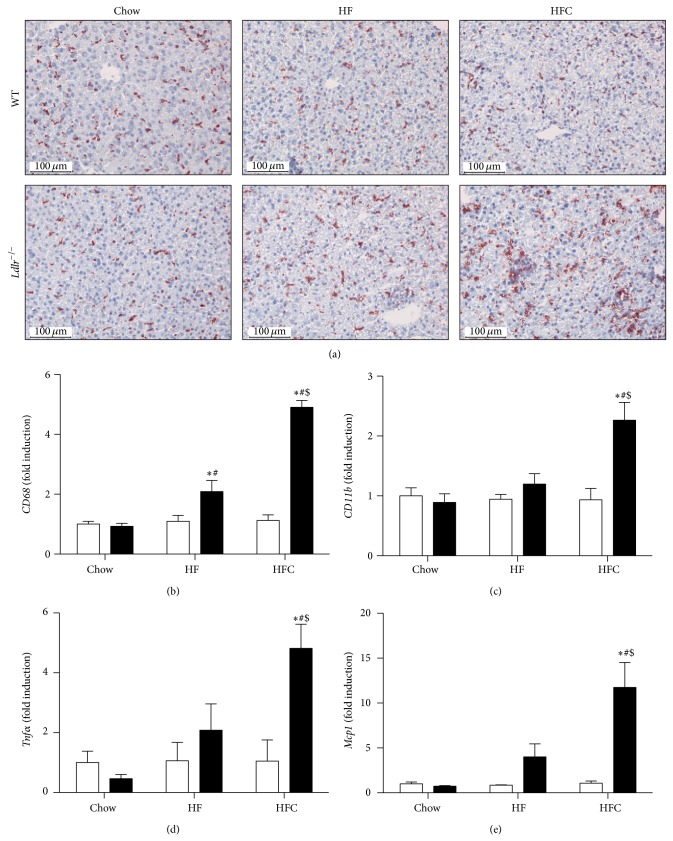
Hepatic inflammation in* Ldlr* ^−/−^ mice fed an HFC diet. (a) Representative pictures of frozen liver sections stained with CD68 were taken from WT and* Ldlr* ^−/−^ mice fed a chow, high-fat (HF), or high-fat cholesterol (HFC) diet (*n* = 5-6). RNA was isolated from liver tissue and the expression of the proinflammatory genes* Cd68* (b),* Cd11b* (c),* Tnfa* (d), and* Mcp1* (e) was determined by real-time PCR and expressed as fold induction (*n* = 5). Data are expressed as means ± SEM for WT mice (white bars) and* Ldlr* ^−/−^ mice (black bars). ^*^ *P* < 0.05 WT versus KO; ^#^ *P* < 0.05 HF, HFC versus chow; ^$^ *P* < 0.05, HF versus HFC.

To validate the degree of hepatic inflammation in WT and* Ldlr* ^−/−^ mice fed a chow, HF, and HFC diet, we performed CD68 immunostaining and measured the expression of the proinflammatory genes* Cd68*,* Cd11b*,* Tnfa*, and* Mcp* in livers of WT and* Ldlr* ^−/−^ mice fed a chow, HF, and HFC diet. As expected,* Ldlr* ^−/−^ mice fed an HFC diet showed an increased staining of CD68 in the liver (Figure 2(a)), indicating an increased number of macrophages in their livers. Being consistent with the histological analysis of the liver,* Ldlr* ^−/−^ mice on an HFC diet showed marked levels of hepatic inflammation compared to HFC-fed WT mice. We observed a 5-fold and a 2-fold increase in the mRNA levels of the macrophage markers* Cd68* and* Cd11b*, respectively (Figures 2(b) and 2(c)), compared to HFC-fed WT mice. In addition, a 5-fold increase in the expression of the cytokine* Tnfa* (Figure 2(d)) and a 10-fold increase in the expression of the chemokine* Mcp1* (Figure 2(e)) were observed in HFC-fed* Ldlr* ^−/−^ mice compared to HFC-fed WT mice. Furthermore, increased* Cd68* expression was also observed in* Ldlr* ^−/−^ mice following 2 weeks of HF feeding compared to HF-fed WT mice (Figure 2(b); *P* < 0.05). However, this HF-diet induced increase in* Cd68* expression was significantly lower compared to* Ldlr* ^−/−^ mice fed an HFC-diet (Figure 2(b)). Moreover, HF feeding did not increase the expression levels of* Cd11b*,* Tnfa*, or* Mcp1 *in the livers of* Ldlr* ^−/−^ mice whereas HFC feeding did (Figures 2(c)–2(e);* Ldlr* ^−/−^ HF versus* Ldlr* ^−/−^ HFC, *P* < 0.05 for all genes).

### 3.3. Absence of Adipose Tissue Inflammation in* Ldlr* ^−/−^ Mice Fed an HFC Diet

**Figure 3:**
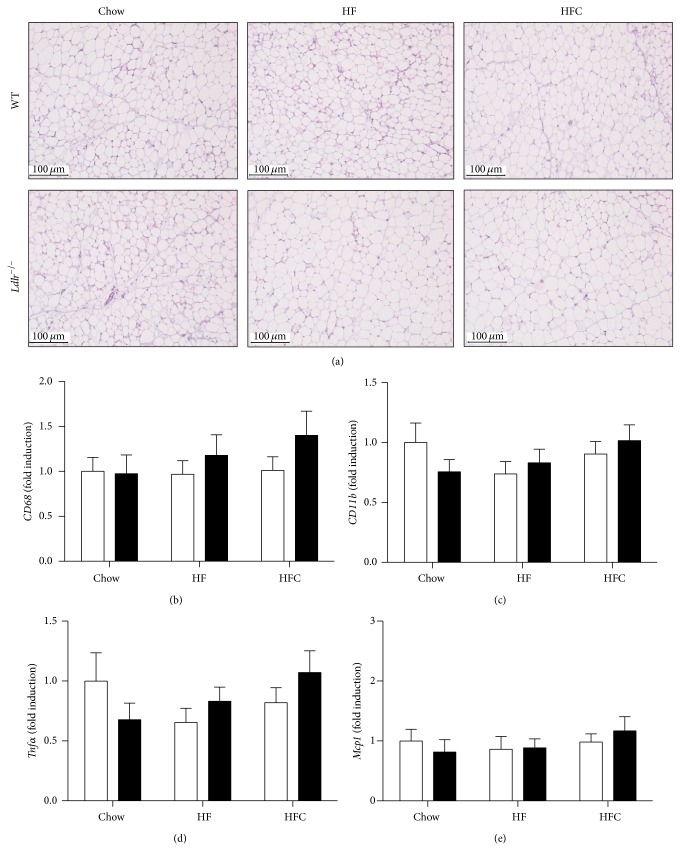
Absence of adipose tissue inflammation in* Ldlr* ^−/−^ mice fed a chow, HF, or HFC diet for 2 weeks. Representative pictures of paraffin-embedded white adipose tissue sections stained with hematoxylin-eosin (a) were taken from WT and* Ldlr* ^−/−^ mice fed a chow, HF, or HFC diet (*n* = 5-6). RNA was isolated from white adipose tissue and the expression of the proinflammatory genes* Cd68* (b),* Cd11b* (c),* Tnfa* (d), and* Mcp1* (e) was determined by real-time PCR and expressed as fold induction (*n* = 11-12). Data are expressed as means ± SEM for WT mice (white bars) and* Ldlr* ^−/−^ mice (black bars).

Despite marked inflammation in the livers of* Ldlr* ^−/−^ mice fed an HFC diet, hematoxylin and eosin staining of white adipose tissue sections did not show signs of inflammation (Figure 3(a)). In addition, no significant changes in the gene expression of the inflammatory markers* Cd68*,* Cd11b*,* Tnfa*, and* Mcp* were found in white adipose tissue (Figures 3(b)–3(e)), confirming the absence of adipose tissue inflammation in* Ldlr* ^−/−^ mice fed any of the given diets.

### 3.4. Hepatic Insulin Resistance in Dyslipidemic* Ldlr* ^−/−^ Mice Is Unrelated to Hepatic Inflammation

**Figure 4:**
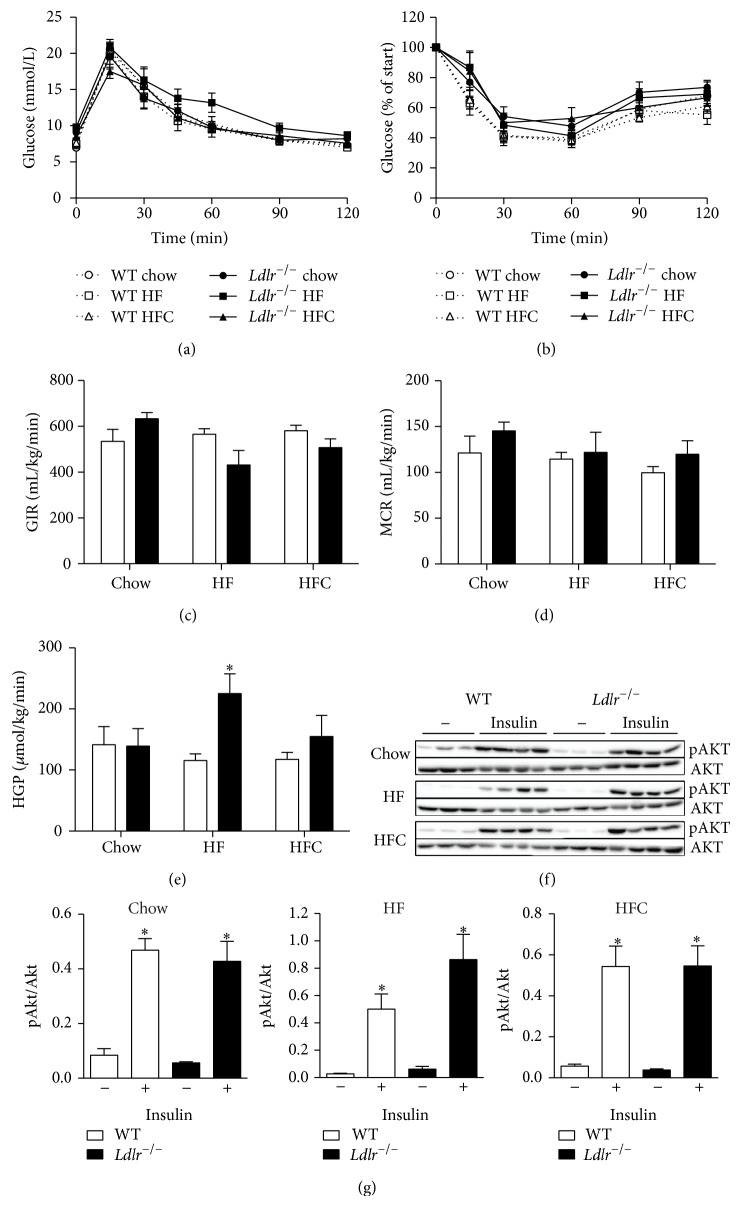
Hepatic inflammation does not induce hepatic insulin resistance in lean* Ldlr* ^−/−^ mice. To assess systemic insulin resistance, we performed an oral glucose tolerance test (a) and an insulin tolerance test (b) in WT and* Ldlr* ^−/−^ mice fed a chow, high-fat (HF), or high-fat cholesterol (HFC) diet (*n* = 5-6). To distinguish between hepatic and peripheral insulin resistance, a hyperinsulinemic-euglycemic clamp was performed during which glucose infusion rate (GIR) (c), metabolic clearance rate (MCR) (d), and hepatic glucose production (HGP) (e) were determined (*n* = 5–7). (f) Phosphorylation status of AKT in liver tissues obtained from WT and* Ldlr* ^−/−^ mice fed a chow, HF, or HFC diet sacrificed 15 min after saline (*n* = 5) or insulin injection (*n* = 7) and determined by Western Blot analysis. Data are expressed as means ± SEM for WT mice (white bars) and* Ldlr* ^−/−^ mice (black bars). ^*^ *P* < 0.05 WT versus KO; ^#^ *P* < 0.05 HF, HFC versus chow; ^$^ *P* < 0.05 HF versus HFC.

To investigate the degree of systemic insulin resistance in dyslipidemic* Ldlr* ^−/−^ mice with or without hepatic inflammation, we performed an OGTT and an ITT in* Ldlr* ^−/−^ mice fed a chow, HF, and HFC diet. The OGTT (Figure 4(a)) and ITT (Figure 4(b)) did not detect differences between the groups, suggesting that 2 weeks of HF and HFC feeding did not induce notable changes in glucose and insulin tolerance between the mice. We next performed a hyperinsulinemic-euglycemic clamp (HIEC) to distinguish between hepatic and peripheral insulin resistance. The glucose infusion rate (GIR; Figure 4(c)) and the metabolic clearance rate of glucose (MCR; Figure 4(d)) did not differ between the mice fed a chow, HF, or HFC diet, confirming the similar glucose curves observed during the OGTT and the ITT. However, we observed a significant increase in hepatic glucose production in* Ldlr* ^−/−^ mice fed an HF diet compared to HF fed WT mice (Figure 4(e)). These findings indicate that* Ldlr* ^−/−^ mice fed an HF diet suffered from hepatic insulin resistance, while peripheral insulin resistance remained unaffected. Hepatic glucose production was not increased in* Ldlr* ^−/−^ mice fed an HFC-diet compared to HFC-fed WT mice (Figure 4(e); ns) and was not worse than* Ldlr* ^−/−^ mice fed an HFC diet (Figure 4(e); ns). Although clear hepatic insulin resistance was observed in* Ldlr* ^−/−^ mice fed an HF-diet, the Ser473 phosphorylation of AKT (Figures 4(f) and 4(g)) in the liver was not affected following insulin stimulation. In fact, no difference was observed in the insulin stimulated AKT response for any of the given diets (Figure 4(g)).

### 3.5. Differences in Hepatic Lipid Accumulation Cannot Explain Hepatic Insulin Resistance

**Figure 5:**
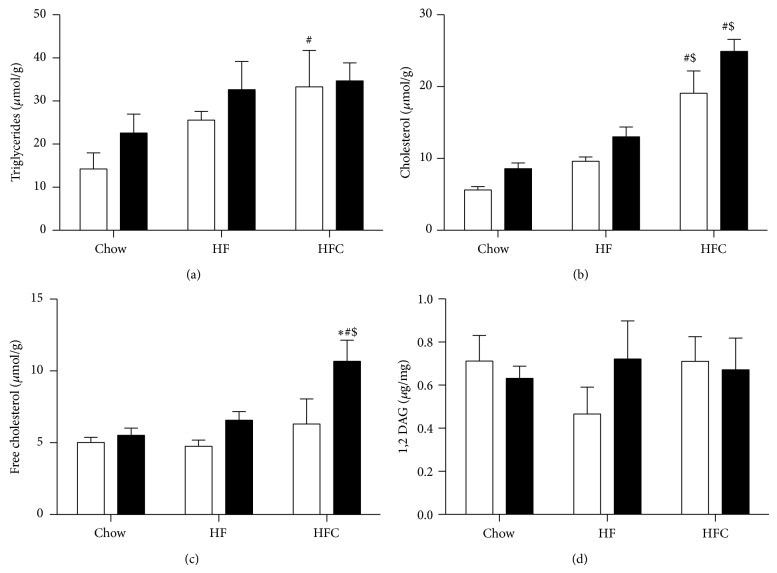
Differences in hepatic lipid accumulation cannot explain hepatic insulin resistance. To assess hepatic lipid accumulation, we measured levels of triglycerides (a) cholesterol (b), free cholesterol (c), and 1,2 DAG (d) in liver tissue of WT and* Ldlr* ^−/−^ mice fed a chow, high-fat (HF), or high-fat cholesterol (HFC) diet. Data are expressed as means ± SEM for WT mice (white bars) and* Ldlr* ^−/−^ mice (black bars) (*n* = 5). ^*^ *P* < 0.05 versus wild type. ^*^ *P* < 0.05, WT versus KO; ^#^ *P* < 0.05 HF, HFC versus chow; ^$^ *P* < 0.05, HF versus HFC.

Since the accumulation of lipid species in the liver has been associated with the development of hepatic insulin resistance [20–22], we measured hepatic lipid accumulation in WT and* Ldlr* ^−/−^ mice fed a chow, HF, or HFC diet for 2 weeks. No difference was observed in hepatic triglyceride accumulation between WT and* Ldlr* ^−/−^ mice fed either diet; however, compared to chow-fed mice triglyceride levels were moderately increased in livers of WT mice (Figure 5(a); WT HFC versus WT chow, *P* < 0.05). As expected, HFC feeding increased total cholesterol levels in both WT and* Ldlr* ^−/−^ mice compared to chow and HF-fed mice and did not differ between the genotypes (Figure 5(b)). Free cholesterol was significantly increased but only in* Ldlr* ^−/−^ mice fed an HFC diet (Figure 5(c)). In particular, DAGs have been associated with the development of hepatic insulin resistance [21]. However, DAG levels were similar in both genotypes and on all diets (Figure 5(d)). In summary, these results suggest that differences in hepatic lipid accumulation cannot account for the hepatic insulin resistance observed in* Ldlr* ^−/−^ mice.

## 4. Discussion

This study was designed to determine the effect of hepatic inflammation on the development of insulin resistance in* Ldlr* ^−/−^ mice, while excluding body weight gain as a confounding factor. Our results show that* Ldlr* ^−/−^ mice develop hepatic insulin resistance within 2 weeks of HF feeding, while peripheral insulin resistance remained unaffected. Our data also show that both systemic insulin resistance and hepatic insulin resistance are not more advanced in* Ldlr* ^−/−^ mice fed an HFC diet, even though these mice had increased levels of hepatic inflammation compared to both chow and HF-fed* Ldlr* ^−/−^ mice. These results illustrate that hepatic insulin resistance can develop prior to alterations in body weight gain. Moreover, our findings suggest that hepatic inflammation induced by dietary cholesterol is not associated with the onset of hepatic insulin resistance during this time frame and indicate that cholesterol-induced hepatic inflammation cannot explain the predisposition towards insulin resistance in these* Ldlr* ^−/−^ mice.

Our results also suggest that dyslipidemia is not causal to the development of hepatic insulin resistance as the degree of dyslipidemia was identical amongst the HF- and HFC-fed* Ldlr* ^−/−^ mice (Figures 1(b) and 1(c)) whereas hepatic insulin resistance was only observed in the HF-fed* Ldlr* ^−/−^ mice (Figure 4(e)). This argues against a causal relationship in the well-established metabolic link between hyperglycemia and dyslipidemia. Indeed, dyslipidemia, at the clinical level, is associated with elevated plasma glucose levels and insulin resistance. Furthermore, patients diagnosed with familial combined hyperlipidemia have an increased incidence of insulin resistance and T2D [23–27]. Moreover, dyslipidemia is an independent predictor for the development of insulin resistance and T2D later in life. Nevertheless, there is a complex genetic regulation and metabolic interplay between lipid and glucose metabolism, as we have recently observed that the genetic predisposition to dyslipidemia is related to lower levels of fasting plasma glucose, HbA1c, and HOMA-IR [28]. Out of the 15 loci that are associated with both lipids and glucose-related traits independently, 8 (CETP, MLXIPL, PLTP, GCKR, APOB, APOE-C1-C2, CYP7A1, and TIMD4) did exert an opposite allelic effect on dyslipidemia and glucose traits [28].

In contrast to several publications that indicate that hepatic inflammation can cause insulin resistance [29, 30], we found that hepatic inflammation did not advance the development of peripheral insulin resistance in female* Ldlr* ^−/−^ mice. This confirms our previous findings in male* Ldlr* ^−/−^ mice fed a 2-week HFC diet [4] suggesting that similar phenomena exist between male and female* Ldlr* ^−/−^ mice in terms of systemic insulin resistance. Whether hepatic insulin resistance is also unrelated to hepatic inflammation in male* Ldlr* ^−/−^ mice remains to be investigated. An explanation for the lack of an effect of hepatic inflammation on insulin resistance may be found in the cell type driving inflammation. Cai et al. described how inflammation was induced by hepatocyte activation of IKK, and this resulted in hepatic and systemic insulin resistance [30]. Though being not assessed in this paper, previous studies have shown that Kupffer cells become foamy in* Ldlr* ^−/−^ mice within 7 days of HFC feeding and may be responsible for the initiation of hepatic inflammation in this model [14, 15]. Kupffer cells are thought to contribute to insulin resistance by the production of proinflammatory cytokines that inhibit insulin signaling in hepatocytes [31]. Nevertheless, there is conflicting evidence for the role of Kupffer cells in hepatic insulin resistance. Some papers report an amelioration of insulin resistance with a depletion of Kupffer cells [32, 33], whereas others show deterioration in insulin resistance [34]. Moreover, depleting Kupffer cells after the induction of insulin resistance has no therapeutic effect on metabolic changes [5]. Therefore, the cell type driving the hepatic inflammation may be important in determining the effect on insulin resistance. Hepatocyte-derived inflammation may be more important than Kupffer cell activation in the development of insulin resistance, highlighting the need for more studies focusing on cell type-specific induction of inflammation.

The lack of an effect of hepatic inflammation on insulin resistance may also reflect a time-dependent effect. A recent paper reported that inflammation was only involved in diet-induced insulin resistance once obesity had been established and not during the onset of obesity [35]. When obesity is established, a crosstalk between adipose tissue and liver may start to play a role. Hence, an HFC diet induces insulin resistance in* Ldlr* ^−/−^ mice only after 24 weeks of HFC feeding, which is presumably caused by adipose tissue inflammation [36]. In another study, the proinflammatory cytokines secreted from adipose tissue were shown to be able to induce insulin resistance in hepatocytes [37]. Hepatic inflammation has been shown to develop within 4 days of HFC feeding in female* Ldlr* ^−/−^ mice [14]. Thus, in our experimental model, hepatic inflammation was present for approximately 1.5 weeks, without increased body weight gain or adipose tissue inflammation (Figures 2(b) and 3(c)) being present. This may not be long enough for hepatic inflammation to inhibit insulin signaling in the liver. Our data indicate that cholesterol-induced hepatic inflammation, in the absence of adipose tissue inflammation, is not enough to induce insulin resistance in hepatocytes. Within this 2-week time frame hepatic insulin resistance may be primarily caused by factors other than hepatic inflammation and dyslipidemia.

A few limitations of our study must be taken into account. While there is no doubt about the hepatic insulin resistance observed in* Ldlr* ^−/−^ fed an HF diet during the HIEC, we were not able to confirm these results by measuring phosphorylation of AKT in the livers of insulin injected mice. This may be explained by the many pathways and molecules that are involved in insulin signaling [38]. Interference with insulin signaling may take place at a different part of the insulin signaling cascade than at the level of AKT. In addition, while we excluded differences in hepatic lipid content that may affect hepatic insulin resistance, we cannot rule out that other changes that could occur in* Ldlr* ^−/−^ mice might contribute to their hepatic insulin resistance. Lack of the* Ldlr* may lead to differences in intracellular signaling cascades that could affect insulin signaling. However, in chow-fed* Ldlr* ^−/−^ mice, we observed no changes in either hepatic or peripheral insulin resistance, indicating that the effects on hepatic insulin resistance are not intrinsic to the* Ldlr* deficiency but are related to the HF-diet intervention in these mice. Moreover, the fact that HFC feeding induced more severe hepatic inflammation and less insulin resistance than did HF feeding does not necessarily indicate that inflammation can be denied as being the cause of insulin resistance in* Ldlr* ^−/−^ mice. The results may thus suggest that cholesterol addition to the HF diet alleviates hepatic insulin resistance in* Ldlr* ^−/−^ mice. As a result, we cannot exclude that deterioration of insulin resistance induced by the hepatic inflammation may have been overcome by the insulin sensitizing effect of cholesterol. Nevertheless, we cannot explain why cholesterol addition to the HF diet confers protection against the development of hepatic insulin resistance in* Ldlr* ^−/−^ mice. This may be related to Kupffer cell activation triggered by the cholesterol in the diet [4], since factors derived from Kupffer cells have been shown to work in a transacting manner to maintain hepatic lipid homeostasis [32]. Further studies are required to understand this effect.

## 5. Conclusions

In conclusion, our data show that neither cholesterol-induced hepatic inflammation nor dyslipidemia is causally related to the development of hepatic insulin resistance in* Ldlr* ^−/−^ mice. As chronic inflammation is considered a causal risk factor predisposing to insulin resistance, our data suggests that inflammation when confined to the liver may not be causal to metabolic dysfunction.
